# *MUC*4 mucin expression in human pancreatic tumours is affected by organ environment: the possible role of TGF*β*2

**DOI:** 10.1038/sj.bjc.6601604

**Published:** 2004-02-03

**Authors:** A Choudhury, N Moniaux, A B Ulrich, B M Schmied, J Standop, P M Pour, S J Gendler, M A Hollingsworth, J-P Aubert, S K Batra

**Affiliations:** 1 Department of Biochemistry and Molecular Biology, University of Nebraska Medical Center, Omaha, NE, USA; 2 Eppley Institute for Research in Cancer and Allied Diseases, University of Nebraska Medical Center, Omaha, NE, USA; 3 Mayo Clinic Scottsdale, Scottsdale, AZ, USA; 4 Unite 560 INSERM, Place de verdun, Lille Cedex 59045, France

**Keywords:** pancreatic adenocarcinoma, mucin, MUC4, orthotopic environment, TGF*β*2

## Abstract

MUC4 is highly expressed in human pancreatic tumours and pancreatic tumour cell lines, but is minimally or not expressed in normal pancreas or chronic pancreatitis. Here, we investigated the aberrant regulation of *MUC4* expression *in vivo* using clonal human pancreatic tumour cells (CD18/HPAF) grown either orthotopically in the pancreas (OT) or ectopically in subcutaneous tissue (SC) in the nude mice. Histological examination of the OT and SC tumours showed moderately differentiated and anaplastic morphology, respectively. The OT tumour cells showed metastases to distant lymph nodes and faster tumour growth (P<0.01) compared to the SC tumours. The *MUC4* transcripts in OT tumours were very high compared to the undetectable levels in SC tumours. The SC tumour cells regained their ability to express *MUC4* transcripts after *in vitro* culture. Immunohistochemical analysis using MUC4-specific polyclonal antiserum confirmed the results obtained by Northern blot analysis. Interestingly, the OT tumours showed expression of *TGFβ2* compared to no expression in SC, suggesting a possible link between MUC4 and *TGFβ2*. The MUC4 expression, morphology, and metastasis of human pancreatic tumour cells are regulated by a local host microenvironment. TGF*β*2 may serve as an interim regulator of this function.

Pancreatic adenocarcinomas are frequently associated with an altered synthesis of mucins (Balague *et al*, 1994; Hollingsworth *et al*, 1994; Kim *et al*, 1999; Andrianifahanana *et al*, 2001). Biochemically, mucins are high-molecular-weight glycoproteins and their polypeptide chains have domains rich in threonine and/or serine, whose hydroxyl groups are in *O*-glycosidic linkage with oligosaccharides. These domains are composed of tandemly repeated sequences that vary in number, length, and amino-acid sequence from one mucin to another (Gendler and Spicer, 1995; Moniaux *et al*, 2001). They are produced mainly by the secretory epithelial cells for the lubrication and protection of ducts and lumen within the human body (Gendler and Spicer, 1995). In all, 14 human mucin genes have been identified, designated as *MUC1-4*, *MUC5B*, *MUC5AC*, *MUC6-8*, *MUC11-12*, *MUC13*, and *MUC16-17* (Gendler *et al*, 1990; Lan *et al*, 1990; Aubert *et al*, 1991; Porchet *et al*, 1991; Bobek *et al*, 1993; Dufosse *et al*, 1993; Gum *et al*, 1994, 1997; Shankar *et al*, 1997; Toribara *et al*, 1997; Williams *et al*, 1999, 2001; Yin and Lloyd, 2001; Gum Jr *et al*, 2002). Based on the structure, mucins are categorised into three distinct forms: membrane spanning (*MUC1*, *MUC3, MUC4*, *MUC12* and *MUC17*), gel forming (*MUC2*, *MUC5AC*, *MUC5B* and *MUC6*), and soluble (*MUC7*) (Moniaux *et al*, 2001). *MUC4* has been cloned from the human trachea and human pancreatic tumours, and the full-length cDNA sequence is known (Nollet *et al*, 1998; Moniaux *et al*, 1999; Choudhury *et al*, 2000a). The NH_2_-terminus of MUC4 is composed of a 27-residue signal peptide and a large domain varying in length from 3285–7285 amino-acid residues as a result of variable number of 16 amino-acid tandem repeat units (VNTR). The COOH-terminus of MUC4 encodes 12 distinct domains that include two cysteine-rich domains, three epidermal growth factor (EGF)-like domains, two regions rich in potential N-glycosylation sites, one hydrophobic transmembrane region, and one short cytoplasmic tail. Nine out of 12 of the C-terminal domains share 60–80% similarity in sequence with the rat sialomucin complex, known as SMC. Sialomucin complex is the rat homologue of human MUC4 and is now known as rat Muc4 (Moniaux *et al*, 1999).

The apomucins *MUC1*, *MUC5B*, *MUC5AC*, and *MUC6* are expressed in the normal pancreas; *MUC1*, *MUC2*, and *MUC4* are upregulated in pancreatic tumours (Andrianifahanana *et al*, 2001), whereas *MUC5B*, *MUC5AC*, and *MUC6* are slightly downregulated. The *MUC4* mucin is expressed at high levels in human pancreatic tumours and tumour cell lines, with an undetectable level in the normal pancreas (Andrianifahanana *et al*, 2001). MUC4 is also expressed by metaplastic ducts and its expression increases with higher grade in pancreatic intraepithelial neoplasias (PanINs) (Swart *et al*, 2002). The human tissues showing an undetectable level of *MUC4* expression are the gall bladder biliary epithelial cells, intrahepatic bile ducts, and the liver (Vandenhaute *et al*, 1997). In contrast, the MUC4 apomucin is expressed in numerous normal human tissues like the stomach, ovary, salivary gland, colon, lung, trachea, uterus, and prostate (Audie *et al*, 1993; Reid and Harris, 1998; Buisine *et al*, 1999; Gipson *et al*, 1999). During foetal development, there is a complex spatiotemporal regulation of the *MUC*4 gene in the gastroduodenal tract and accessory digestive glands.

The membrane-associated mucins rat *Muc4* and *MUC1* have been reported to play a role in tumour progression and metastasis (Gendler and Spicer, 1995; Komatsu *et al*, 1997, 2000; Kim *et al*, 1999). The implantation of human tumour cells in the pancreas of nude mice (orthotopic (OT) implantation) has proved to be useful in studying the progression of pancreatic cancer *in vivo* (Marincola *et al*, 1989). The organ microenvironment has been shown to influence the physiological properties of the tumour cells in the production of degradative enzymes and the regulation of *mdr1* mRNA and P-glycoprotein expression (Fidler *et al*, 1994). The survival and growth of a particular tumour cell are significantly affected by the local milieu provided by a particular organ environment (Fidler *et al*, 1994).

In the present study, the influence of the local host microenvironment on the expression of the *MUC4* transcript and protein was examined for the first time. Using human pancreatic adenocarcinoma cell lines, CD18/HPAF and SW1990, human pancreatic tumour xenografts were developed at the OT and subcutaneous (SC) sites of the nude mouse. The morphologically differentiated invasive OT tumours demonstrated a high level of expression of *MUC4* mRNA and protein compared to undetectable levels in poorly differentiated SC tumours. However, the *in vitro* culture of SC tumour cells resulted in the expression of *MUC4* transcripts comparable with its expression level in the parental cell line CD18/HPAF. Paracrine stimulation by growth factors and cytokines has been demonstrated to be one of the mechanisms responsible for the organ preference and proliferation of the tumour cells. The MUC4-expressing OT tumours also showed transforming growth factor (*TGF*)*β2* expression. The study suggests that the site of pancreatic tumour growth *in vivo* strongly influences *MUC4* and *TGFβ2* expression, tumour morphology, and invasiveness of CD18/HPAF cells.

## MATERIALS AND METHODS

### Animals

Female athymic mice (nu/nu) (6–8 weeks) were obtained from Charles River (Wilmington, MA, USA). The mice were housed in laminar flow cabinets under specific pathogen-free conditions. The University of Nebraska Medical Center Institutional Animal Care and Use Committee approved animal protocols used in this study (IACUC #97-069-03), which comply with the Public Health Service Policy on the Humane Care and Use of Laboratory Animals.

### Tumour cell line and tumour cell culture

The CD18/HPAF cell line used in the study was originally derived from the parental heterogeneous HPAF pancreatic tumour cell line by a limiting dilution technique (Metzgar *et al*, 1982; Kim *et al*, 1989). Cells were cultured in Dulbecco's modified Eagle medium (DMEM) containing 10% foetal bovine serum (FBS) and penicillin–streptomycin 200 U ml^−1^ (Life Technologies Inc., Grand Island, NY, USA). The SW1990 cell line was established from spleen metastasis of grade II pancreatic adenocarcinoma derived from the exocrine pancreas (Kyriazis *et al*, 1983). Cells were culture in leibovitz's L-15 medium with 10% FBS and penicillin–streptomycin 200 U ml^−1^ (Life Technologies Inc.) at 37°C without CO_2_. For *in vivo* injections, cells were harvested from subconfluent cultures by treatment with 0.05% trypsin and 0.53 mM EDTA (trypsin-EDTA solution; Life Technologies Inc.) and resuspended in Hank's balanced salt solution (HBSS) for injection. Only single-cell suspensions with >90% viability were used for injection.

A portion of tumour tissue, obtained 2 weeks after implantation of the CD18/HPAF cells into the pancreas or the SC tissue of the nude mice, was placed in a 10% DMEM medium and minced finely with a scalpel. The medium containing the tissue pieces was centrifuged and the supernatant containing the floating fat tissue was removed. The tissue pellet was treated with DMEM supplemented with collagenase P (3.75 mg ml^−1^ medium) at 37°C for 15 min. The digestion of the tissue was terminated by adding 10% DMEM. After washing the tissue three times in DMEM medium, tissue fragments were seeded into six-well plates and incubated at 37°C in a humidified atmosphere of 5% CO_2_ in air. After 24 h, tumour cells began to migrate out from the tissue pieces into the surrounding areas. The wells became subconfluent at day 5 and were trypsinised with Trypsin-EDTA solution twice for different time periods: first for 1 min to detach and remove the fibroblasts and second for 5 min to harvest the tumour cells. Cells were washed and seeded in flasks containing 10% DMEM medium.

### OT and ectopic implantation of tumour cells

Tumour cells were harvested from the culture flasks by trypsinisation in EDTA solution, and were washed by centrifugation in a serum-containing medium. After being washed twice in PBS pH 7.4 (Life Technologies Inc.), they were resuspended in the same buffer at a concentration of 10 × 10^6^ cells ml^−1^. Mice were anaesthetised with 100–200 mg kg^−1^ ketamine and 5–16 mg kg^−1^ of xylazine. A volume of 50 *μ*l of cell suspension (10 × 10^6^ cells ml^−1^) was injected into different tissues like the pancreas, submandibular gland (SMG), ovary, stomach, and SC site. After the injection, the organ was returned to the correct position and the abdomen was sutured using chromic catgut. The skin was closed with metal clips, which were removed 10 days later. The SC injection was performed using 50 *μ*l of cell suspension at a site on the back between the scapulae. After implantation, mice were inspected twice a week. Tumour formation was checked twice a week in the first 2 weeks and daily thereafter. Tumour-bearing mice were killed when an intra-abdominal mass measuring ∼2 cm in diameter was palpated. To assess the tumour dissemination pattern, in each group at least four mice were kept alive until they were moribund. After sacrifice, primary tumours and the metastatic tumours were weighed, measured, and cut into small fragments. These fragments were processed for immunohistochemistry (IHC) or RNA isolation, or were kept in tissue culture medium and processed to obtain a cell line, as described in the previous section.

### Isolation of RNA and Northern blotting

The total cellular RNA from the normal human pancreas, human pancreatic xenografts, and CD18/HPAF cell line was isolated by the guanidine isothiocyanate and cesium chloride (CsCl) cushion ultracentrifugation method (Chirgwin *et al*, 1979). The Northern blots were performed as described previously (Choudhury *et al*, 2000b).

### Immunohistochemical examinations

Xenographic tumour tissues were fixed in 10% buffered formalin and embedded in paraffin. Tumour sections (5 *μ*m) were assayed for MUC4 apomucin by using a modification of the previously described ABC immunohistochemical method (Pour *et al*, 1993; Batra *et al*, 1995). Briefly, tissue sections were deparaffinised in xylene, rehydrated in graded ethanol, and treated for 20 min with 0.3% H_2_O_2_/methanol to block endogenous peroxidase. The sections were blocked with normal goat serum for 1 h, followed by incubation at 4°C overnight with either anti-MUC4 rabbit antiserum raised against 16 amino-acid tandem repeat peptide (Ser Thr Gly Asp Thr Thr Pro Leu Pro Val Thr Asp Thr Ser Ser Val) or pre-immune rabbit serum as a negative control. The specificity of the antisera generated against the tandem-repeat peptide in staining pancreatic tumour cells was evaluated as described earlier (Choudhury *et al*, 2000b).

### Semiquantitative reverse transcription–polymerase chain reaction (RT–PCR)

Total RNA (0.5 *μ*g) from the tumour tissue or cell lines was reverse transcribed using the first-strand cDNA synthesis kit (Perkin-Elmer, Branchburg, NJ, USA) and oligo d(T) primers, according to the manufacturer's instructions. Oligonucleotide primers to the nontandem repeat region of *MUCs 1*, *2*, *3*, *4*, *5AC*, *5B*, *6*, *7*, *TGFβ1*, and *TGFβ2* are designed from the published sequences in the GenBank, as described earlier (Choudhury *et al*, 2000b). The mucin genes and *TGFβ* were coamplified with the same *GAPDH* primers. Amplifications were performed in a programmable thermal controller (PTC-100, MJ Research, Inc., Watertown, MA, USA). PCR amplification reactions were described previously (Andrianifahanana *et al*, 2001; Choudhury *et al*, 2000b). For convenience, the corrected densitometric scores for different products were categorised in three different ranges: high value (+++), moderate value (++), and weak value (+). Each value was determined as the mean of four densitometry readings.

## RESULTS

### *In vivo* tumorigenicity and metastatic behaviour of CD18/HPAF cells

CD18/HPAF cells were implanted orthotopically (OT) or ectopically (SC) in nude mice. After 7 days, at any given time point, the extent of tumour growth was higher at OT compared to SC sites. At 20 days after injection, the OT tumour volume was found to be 2.5-fold higher as compared to the SC tumours, reflected in the tumour weight, as shown in Table 1

**Table 1:** Tumorigenicity and production of spontaneous metastases in CD18/HPAF cells

**Injection site**^a^	**Latent period**^b^	**Tumour volum**e^c^	**Tumour weigh**t^d^	**Tumorigenicit**y^e^	**LN metastase**s^f^
SC	11±0.64	980±125.53	1.0±0.42	9/10	0/4
OT	7±0.40	2552±582.97	1.8±0.52	9/10	4/4

a 10 × 10^6^ viable tumour cells were injected into the pancreas or subcutis of two groups of 10 mice/group.

b Days post-injection (±SE), when the tumours could be palpated.

c Tumour volume (mm^3^±s.e.) on day 20 Tumour size was measured with a caliper.

d Average of the weight (in grams±s.e.) of the tumours isolated from different mice.

e Number of mice with tumours/number of injected mice.

f Lymph node (LN) metastases include mediastinal lymph node, mesenteric lymph node, iliac lymph node, and inguinal lymph node metastases.

. Among the six mice bearing OT tumours, two showed extensive invasion of the stomach and duodenum, and three showed regional invasion of the stomach and duodenum. Four mice from each group (OT and SC) were killed after 30 days when they became moribund, and were dissected to examine the sites of metastasis. Tumours of CD18/HPAF cells in pancreas (OT tumours) showed a high incidence of metastases to regional lymph nodes (LNs) and distant metastasis to mediastinal LNs and mesenteric LNs. In contrast, the SC tumours were confined to the site of injection and none of the mice harbouring these tumours showed detectable signs of metastases (Table 1). None of these tumours (OT or SC) showed any signs of necrosis.

Similar results were obtained using another pancreatic tumour cell line, SW1990, where a significant difference in tumour volume (*P*<0.01) was observed for the OT tumour 6182+1003 mm^3^ compared to SC tumour 1774+844 mm^3^. The OT tumours metastasised to LNs in all animals compared to no metastasis in the SC tumours.

### Expression of *MUC4* mRNA by OT and SC tumours

**Figure 1:**
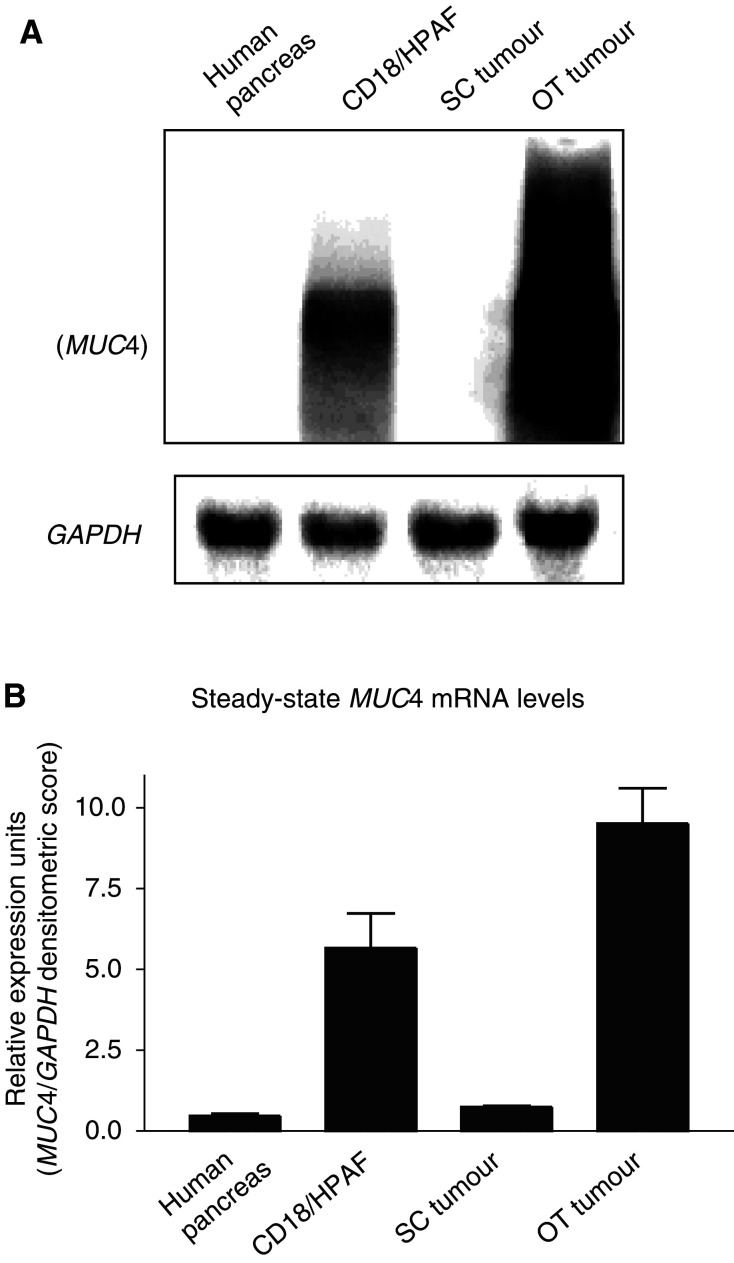
Northern blot of total cellular RNA (20 *μ*g) separated in a 1% agarose/formaldehyde gel from the normal human pancreas tissues, CD18/HPAF cultured cells, and the CD18/HPAF cells grown as SC and OT tumours. (**A**) Blot was probed with a ^32^P-labelled *MUC4* tandem repeat cDNA probe, and the same membrane was stripped and hybridised with a *GAPDH* cDNA probe. (**B**) Densitometric values (±s.e.) for the bands above in three different experiments were determined by using Molecular Dynamics ImageQuant software program. Values obtained for the *MUC4* smear were divided by the densitometic values for the *GAPDH* band.

We further analysed the status of *MUC4* transcripts in the tumours that are generated in two different host environments. Total RNA isolated from the tumour cell line (CD18/HPAF), tumour tissues, and normal human pancreas was fractionated on agarose gel electrophoresis, Northern blotted, and probed with a *MUC4* tandem repeat cDNA probe. As reported in our previous study (Choudhury *et al*, 2000b), the *MUC4* cDNA probe hybridised to a large-sized transcript (∼26.5 kb) in CD18/HPAF cells, and showed a smear ranging from 10 to 29 kb in the OT tumours on Northern blot (Figure 1

). The conceptual expected transcript size of the *MUC4* in HPAF cells will be ∼26.5 kb (Choudhury *et al*, 2000a). OT tumours showed an MUC4 transcript similar or little higher than the parental cell line CD18/HPAF (Figure 1). On the other hand, *MUC4* was not detected in the SC tumour, and the value showed in Figure 1 is the background. The *MUC4* mRNA expression in normal human pancreas was below the background level.

### Histology and MUC4 protein expression in tumours

**Figure 2:**
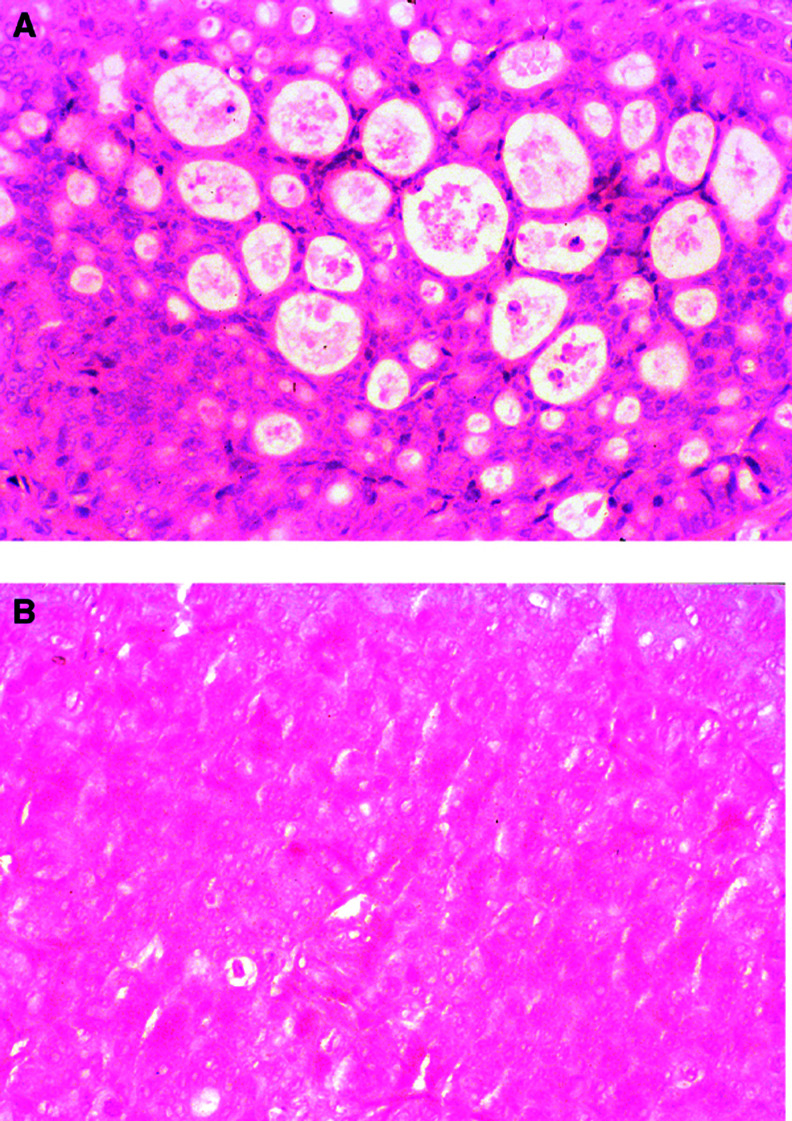
Tumour of CD18/HPAF cells grown in nude mice. (**A**) OT tumour showing a moderately differentiated tumour with glandular structures filled with mucin. (**B**) The same cells grown in SC tissue, showing an amorphous mass of tumour cells with no signs of differentiation. Original × 32.

We studied the tumour histology of the *MUC4*-expressing OT as well as the tumours showing undetectable *MUC4* expression, and were interested to see if there is any correlation with the tumour morphology. Histopathological examination of the tumour tissues stained with haematoxylin and eosin revealed well-developed duct formation and cellular polarisation in the OT tumour (Figure 2A

). In contrast, the SC tumour sections showed an amorphous mass of cells with very little development of ducts in the tumours, and the cells were anaplastic. The tumour cells lacked cellular polarisation and, therefore, did not form luminal spaces (Figure 2B).

**Figure 3:**
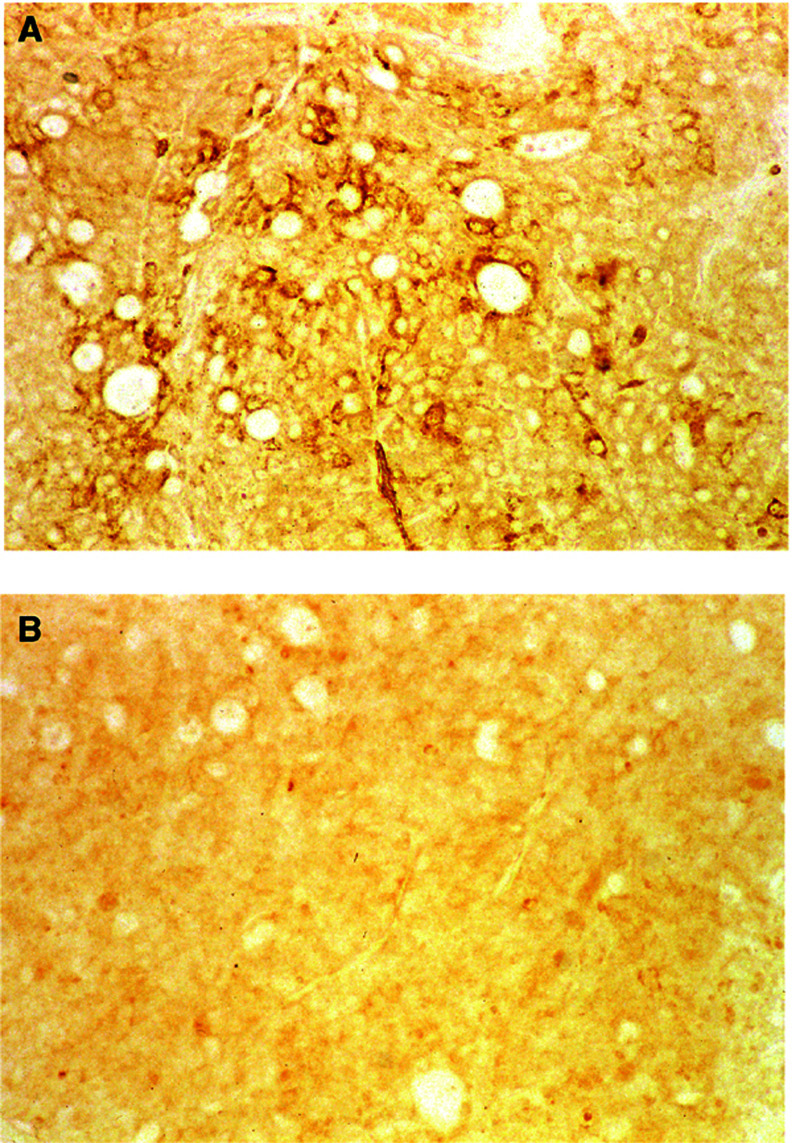
Immunohistochemical staining of CD18/HPAF cells grown in the pancreas of the nude mouse (**A**) and in SC tissues (**B**). The CD18/HPAF tumours in the pancreas show immunoreactivity to anti-*MUC4* antiserum (1 : 100 dilution (**A**)), whereas the pancreatic tissue of the nude mouse remains unstained, as do the tumours grown in SC tissue (**B**). Original × 50 (**A**, **B**).

The MUC4 protein expressions in OT and SC tumour sections were determined by IHC, using a rabbit polyclonal antiserum raised against MUC4 (Choudhury *et al*, 2000b). The immunoreactivity of the anti-MUC4 antibody was seen in OT tumour sections (Figure 3A

), but not in SC tumour sections (Figure 3B). The positive staining in the OT tumour section was specifically blocked by pre-incubation of the MUC4-antiserum with the tandem repeat peptide (data not shown). The control sera (i.e., the pre-immune sera) did not show reactivity with any tumours. The OT tumours showed metastases to the LNs (Table 1). The metastasised LN tumours showed morphology and MUC4 staining similar to that observed in the OT tumours (data not shown).

### *In vitro* expression of MUC4 mRNA by OT and SC tumour cells

**Figure 4:**
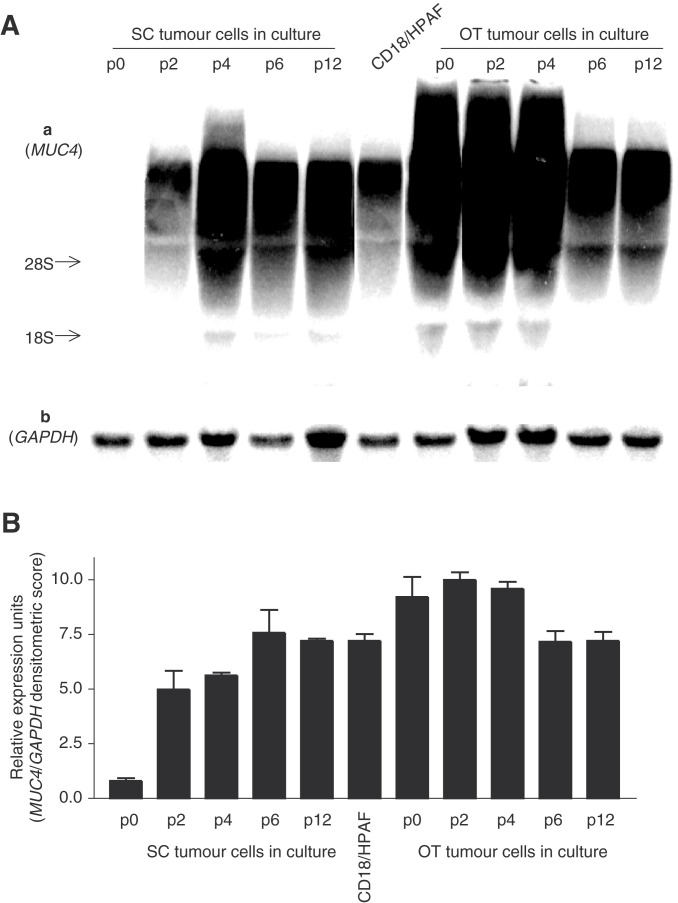
(**A**) Northern blot of total cellular RNA (20 *μ*g) extracted from SC tumour cells, OT tumour cells at different passages and CD18/HPAF cell line, separated in a 1% agarose/formaldehyde gel. (a) Probed with ^32^P-labelled *MUC4* tandem repeat cDNA probe. (b) The same membrane as shown in (a), probed with a *GAPDH* cDNA probe. (**B**) Densitometric values (±s.e.) for the bands in three different experiments were determined by using Molecular Dynamics ImageQuant software program. Values obtained for the *MUC4* smear were divided by the densitometric values for the *GAPDH* band.

To answer the question if there was a clonal expansion of non-MUC4-expressing cells in the SC tumours (showing undetectable levels of MUC4), we isolated and cultured cells from the SC tumours, and studied the MUC4 expression. In SC tumour cells cultured *in vitro*, *MUC4* mRNA expression appeared gradually and increased from passage 2 to 6, with an expression level similar to MUC4 in the CD18/HPAF parental cell line in later passages (Figure 4

). OT tumour cells in culture showed a transient decrease in the level of *MUC4* transcripts and also exhibited a level comparable to MUC4 in CD18/HPAF (Figure 4) in the later passages.

### TGF*β* expression in tumour cells, OT and SC tumours

**Figure 5:**
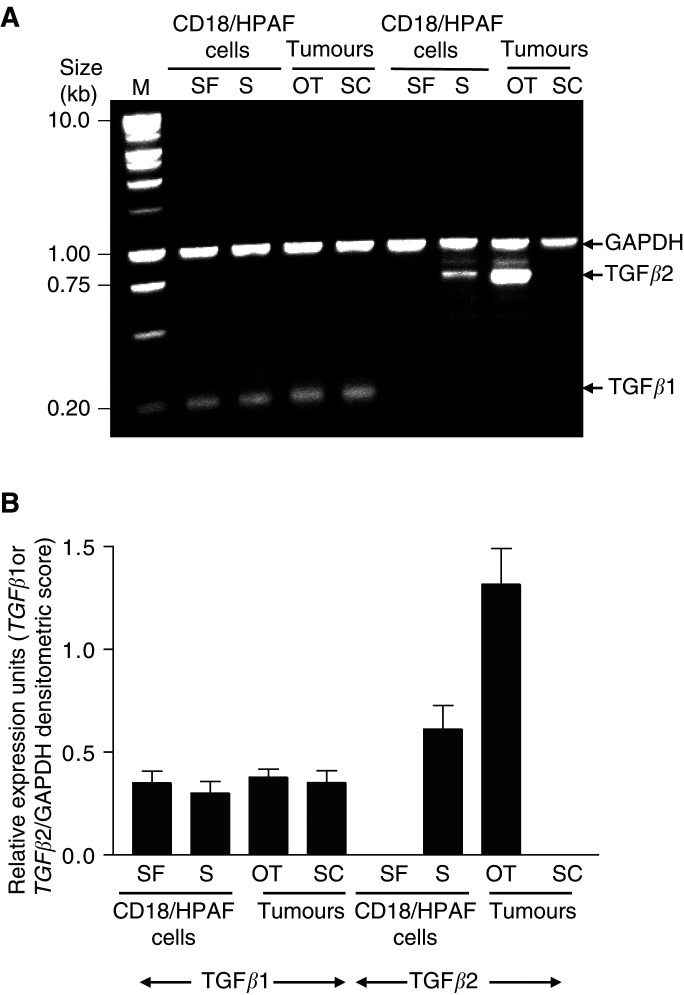
(**A**) Analysis of *TGFβ1* and *TGFβ2* expression in CD18/HPAF cells, OT tumours, and SC tumours. Total RNA was isolated; *TGFβ* and *GAPDH* mRNA are coamplified in each reaction by RT–PCR. (**B**) The band intensity of the amplified products was quantified for each sample using the gel expert™ 3.5 software suite. The densitometric values (±s.e.) for the bands in three different experiments were calculated for a gene-specific product and *GAPDH* for each reaction. The value for a gene-specific product is expressed per unit of *GAPDH* to account for any differences in the starting amounts of RNA. OT, orthotopic tumour; SC, subcutaneous tumour; S, serum; SF, serum-free.

Previously, we demonstrated a positive correlation in the expression of *MUC4* and *TGFβ2* transcripts (Choudhury *et al*, 2000b). For this analysis, the expression of *TGFβ1* and *TGFβ2* was studied by RT–PCR using total RNA isolated from CD18/HPAF cells and OT and SC tumours (Figure 5

). We found that OT tumours showed *TGFβ2* expression with a range two-fold higher than the parental cell lines CD18/HPAF. However, the SC tumour samples showed no undetectable *TGFβ2* expression. Sometimes, a very low level of TGF*β*2 was detected in SC tumours (two out of the six) without expression of MUC4 (data not shown). On the other hand, the expression of *TGFβ1* was found to be similar in tumours (OT and SC) and the CD18/HPAF cells.

As a control, another cell line, SW1990, was used to validate the results. The SW1990 line was implanted in OT and SC sites in nude mice. Expression of MUC4 as well as TGF*β*2 was investigated. The SW1990 OT exhibited a high level of MUC4 and TGF*β*2, as compared to the SW1990 parental line or the SW1990 SC (data not shown).

### Expression of mucin genes in normal human pancreas, CD18/HPAF cell line, OT and SC tumours

To obtain a comparative picture of *MUC4* expression along with other mucin genes, RT–PCR amplification was performed using mucin gene-specific and *GAPDH* primers designed from the published sequences in the GenBank. A comparison of mucin gene expression in the normal human pancreas tissue, pancreatic tumour cell line (CD18/HPAF), and tumour tissues (OT and SC) is shown in Table 2

**Table 2:** RT–PCR expression analysis of mucin genes expression

**Sample**	**MUC1**	**MUC2**	**MUC3**	**MUC4**	**MUC5AC**	**MUC5B**	**MUC6**	**MUC7**
CD18/HPAF	+++	+	−	+++	+/−	++	−	−
OT tumour	+++	−	−	+++	+	++	−	−
SC tumour	+++	−	−	−	+	++	+/−	−
Pancreas	++	−	−	−	+/−	++	++	−

Three orthotopic (OT), three subcutaneous (SC), and seven normal human pancreas were analysed. +++, high; ++, moderate; +, low; −, undetectable; ND, not determined.

. Consistent with the Northern blot and IHC, RT–PCR showed no expression of *MUC4* in the normal human pancreas and the SC tumours, whereas a high level of *MUC4* expression was found in the CD18/HPAF cell line and the OT tumours. The expression of *MUC1* and *MUC5B* appeared similar in all the samples. *MUC2* was detected only in the tumour cell line, but not in the normal human pancreas and tumour samples. The expression of *MUC5AC* was weak in OT and SC tumours, with traces in the cell line and normal human pancreas. *MUC6* was detected at a high level only in the normal pancreas. *MUC3* and *MUC7* mRNA expression was not detected in the CD18/HPAF cell line and tumour samples. The positive controls (as mentioned in the Materials and methods section) for *MUC2*, *MUC3*, *MUC5AC*, *MUC5B*, and *MUC7* showed mucin expression. Among eight mucin genes analysed, *MUC4* was the only gene that showed high levels of expression in OT tumours, with no detectable expression in the normal pancreas or SC tumours. For PCR analysis, primers were designed in the non-tandem repeat regions of the human mucin genes. The amplified PCR products for each mucin gene showed the expected size with 100% sequence identity to the corresponding human sequences, thereby ruling out the possibility of amplification of the mouse Muc4.

## DISCUSSION

The MUC4 mucin exhibits a pancreatic tumour-associated expression, with no detectable expression in a normal pancreas (Balague *et al*, 1994; Hollingsworth *et al*, 1994; Kim *et al*, 1999; Choudhury *et al*, 2000a; Andrianifahanana *et al*, 2001). Based upon the structural information, MUC4 has been proposed to exist as a heterodimeric molecule consisting of a large mucin-type subunit and a membrane-anchored subunit with three EGF-like domains (Moniaux *et al*, 1999; Choudhury *et al*, 2000a). It has been proposed that the MUC4 mucin may have growth factor-like properties, because its rat homologue (rat Muc4) is referred to interact with the oncogene p185^neu^ (Carraway *et al*, 1999). Overexpression of SMC (rat Muc4) has been shown to promote tumour growth in primary tumours and has resulted in metastasis (Komatsu *et al*, 2000).

In the present study, the effect of the host local environment on MUC4 expression was examined in nude mice. The clonal human pancreatic cancer cell line CD18/HPAF that expresses a high level of *MUC4* mRNA (Choudhury *et al*, 2000b) was used for generating human pancreatic xenografts at different sites in nude mice. We observed significantly higher tumour growth (*P*<0.01) rates after implantation at OT sites compared to SC sites in nude mice. In addition, the OT tumours also showed a high incidence of metastasis to regional LNs, and distant metastasis to mediastinal LNs and mesenteric LNs. A high level of MUC4 transcripts and proteins was observed in the human pancreatic tumour xenografts at an OT site, a site analogous to human pancreas that does not show *MUC4* expression, compared to SC sites. When tumours were generated at other MUC4-expressing sites in nude mice like SMG and stomach, these tumours also showed equivalent levels of MUC4 (unpublished result). Pancreas, SMG, and stomach, being physiologically active organs that are well perfused compared to the SC environment, have spare vasculature. The MUC4-expressing cells, when grown in the well-vascularised site (OT), revealed a high level of MUC4 expression, as compared to a less-vascularised (SC) environment. The observation suggests a role of serum factors in regulating the MUC4 expression, or there may be a clonal expansion of a non-*MUC4*-expressing cell type in the SC tumours. To answer these questions, we harvested SC tumour cells and cultured them *in vitro*. The *in vitro* culture of SC tumour cells returned the expression of *MUC4* transcripts to the parental cell line level, further suggesting a role of serum factor(s) in regulating *MUC4* expression. Our earlier study also demonstrates a serum-dependent increase in MUC4 expression in human pancreatic tumour cells (Choudhury *et al*, 2000b).

Further, histological examination of the tumours revealed the OT tumours as moderately differentiated, whereas the SC tumours were poorly differentiated, suggesting that the expression of *MUC4* could be influenced by the differentiation grade of tumours. We have made similar observations on a panel of pancreatic tumour cell lines, where a majority of differentiated adenocarcinomas showed higher levels of *MUC4* transcripts compared to cell lines derived from poorly differentiated adenocarcinomas (Hollingsworth *et al*, 1994; Choudhury *et al*, 2000a; Andrianifahanana *et al*, 2001).

The lack of detectable expression of *MUC4* in SC tumours could also be due to paracrine regulation from the surrounding tissue environment that may be blocking the transcription of *MUC4*. Paracrine stimulation by growth factors and cytokines has been demonstrated to be one of the mechanisms responsible for the organ preference and proliferation of the tumour cells. In the human colon carcinoma cell line, paracrine stimulation by a soluble factor from human colon connective tissue was involved in inducing the expression of the MUC1 mucin *in vitro* (Irimura *et al*, 1990). Cytokine-like tumour necrosis factor-*α*, interleukins, and EGF have been shown to be involved in the regulation of mucin gene expression (Dabbagh *et al*, 1999; Longphre *et al*, 1999; Takeyama *et al*, 1999; Kim *et al*, 2000; Smirnova *et al*, 2000). One of the cytokines (i.e. TGF-*β*) showed an increased expression in many advanced human cancers (Gorsch *et al*, 1992; Gold *et al*, 1994) including pancreatic cancer (Friess *et al*, 1993a, 1993b). A TGF*β*2-dependent increase in MUC4 expression in pancreatic adenocarcinoma (Choudhury *et al*, 2000b) and elevated levels of *TGFβ2* transcripts in MUC4-expressing OT tumours suggest the involvement of this cytokine in MUC4 regulation by autocrine and/or paracrine manner in CD18/HPAF tumours. Nevertheless, the expression of MUC4 is also regulated by TGF*β*2, post-translationally and post-transcriptionally in normal and mammary adenocarcinoma cells, respectively (Price-Schiavi *et al*, 1998, 2000).

Earlier studies have shown that the organ environment can influence tumorigenesis; production of degradative enzymes; melanin and angiogenic molecules; induction of terminal differentiation; level of P-glycoprotein associated with the multiple drug-resistance phenotype; and IL-8 expression (Hart and Fidler, 1980; Price *et al*, 1988; Nakajima *et al*, 1990; Staroselsky *et al*, 1990; Fabra *et al*, 1992; Wilmanns *et al*, 1992; Radinsky *et al*, 1994; Gutman *et al*, 1995). The influence of the organ environment on the growth of tumour cells was originally proposed in Paget's hypothesis of the ‘seed and the soil’ (Paget, 1989).

In summary, we believe that this is the first study showing *in vivo* regulation of human MUC4 expression in pancreatic tumours. The expression of MUC4 was high in moderately differentiated tumours, with undetectable levels in poorly differentiated SC tumours. The OT tumours also showed metastases not only to the regional but also to the distant LNs. The SC tumour cells, when cultured *in vitro*, showed *MUC4* expression, suggesting a role of serum factor(s) in its regulation. Our results also indicated a direct correlation between the MUC4 expression and the levels of *TGFβ2* transcripts in the CD18/HPAF tumours, as well as in CD18/HPAF cells *in vitro*, as described earlier (Choudhury *et al*, 2000b). These results reveal that the local host environment regulates the MUC4 expression in pancreatic tumours (CD18/HPAF) under *in vivo* conditions.
